# Patients with schizophrenia activate behavioural intentions facilitated by counterfactual reasoning

**DOI:** 10.1371/journal.pone.0178860

**Published:** 2017-06-06

**Authors:** Fernando Contreras, Auria Albacete, Cristian Tebé, Bessy Benejam, Agnes Caño, José Manuel Menchón

**Affiliations:** 1 Department of Psychiatry, Bellvitge University Hospital- IDIBELL, L'Hospitalet de Llobregat, Spain; 2 Department of Clinical Sciences, School of Medicine, University of Barcelona, L'Hospitalet de Llobregat, Spain; 3 Carlos III Health Institute, Centro de Investigación Biomédica en Red de Salud Mental (CIBERSAM), Barcelona, Spain; 4 Statistical Advisory Service, Bellvitge Biomedical Institute-IDIBELL, L'Hospitalet de Llobregat, Spain; 5 Faculty of Medicine and Health Sciences, University Rovira i Virgili, Tarragona, Spain; 6 Department of Psychology, University of Girona, Girona, Spain; Hospital Benito Menni, SPAIN

## Abstract

Previous research has associated schizophrenia with an inability to activate behavioural intentions facilitated by counterfactual thinking (CFT) as a step to improving performance. Consequently, these findings suggest that rehabilitation strategies will be entirely ineffective. To extend previous research, we evaluated the influence of CFT in the activation of behavioural intentions using a novel sequential priming paradigm in the largest sample of subjects explored to date.

**Method:**

The main variables assessed were: answer to complete a target task (wrong or correctly), and percentage gain in the reaction time (RT) to complete a target task correctly depending on whether the prime was a counterfactual or a neutral-control cue. These variables were assessed in 37 patients with schizophrenia and 37 healthy controls. Potential associations with clinical status and socio-demographic characteristics were also explored.

**Results:**

When a counterfactual prime was presented, the probability of giving an incorrect answer was lower for the entire sample than when a neutral prime was presented (OR 0.58; CI 95% 0.42 to 0.79), but the schizophrenia patients showed a higher probability than the controls of giving an incorrect answer (OR 3.89; CI 95% 2.0 to 7.6). Both the schizophrenia patients and the controls showed a similar percentage gain in RT to a correct answer of 8%.

**Conclusions:**

Challenging the results of previous research, our findings suggest a normal activation of behavioural intentions facilitated by CFT in schizophrenia. Nevertheless, the patients showed more difficulty than the controls with the task, adding support to the concept of CFT as a potential new target for consideration in future therapeutic approaches for this illness.

## Introduction

Counterfactual thinking (CFT) is a specific type of conditional reasoning manifested as an almost automatic mental representation of alternatives to past events, especially triggered by negative occurrences [1]. These thoughts take the form of “if only” conditional propositions and have an impact on how individuals find meaning in the events that affect them [2]. For instance, in the fictional scenario where John has failed an important test, he might automatically generate a counterfactual thought like *If I had studied harder*, *I could have passed the test*.

As far as the function of CFT is concerned, it seems to play an important role in supporting adaptive behaviour by enabling us to learn from past experiences [3], by modulating emotional states [4], promoting creativity [5] and supporting future planning and prediction [6]. CFT also seems to be related to specific cognitive biases such as the hindsight bias—enhancing memory distortions that contribute to suboptimal decision-making [7]—and to Theory of Mind (ToM) deficits involved in the development of false belief [8].

Thus, although it may sometimes lead to bias, CFT coordinates daily behaviour via course correction, goal cognition, behavioural regulation and performance improvement [3]. For this reason, in 2008, authorities in this field such as Epstude and Roese proposed a functional theory of CFT based on the content-specific pathway concept originally framed in Gollwitzer and Moskowitz’s research on how goals influence actions [3,9]. According to this theory, CFT would promote behavioural change following a regulatory sequence divided into three links or steps: following John’s fictional scenario, (1) the recognition of a problem that automatically activates the generation of CFT (*I should have studied harder*, Step 1); (2) the activation of behavioural intentions for future similar problems/scenarios (*next time*, *I will study harder*, Step 2); (3) the implementation of corrective behaviours in similar future scenarios (*actually studying harder for the next test*, Step 3). Indeed, previous research has already documented the occurrence of these three steps in the general population [6,10,11].

Cognitive impairment has been endorsed as a core feature of schizophrenia in a growing body of studies. This impairment affects several cognitive domains (with a magnitude of moderate to severe) [12,13], and is already present in the early stages of the disorder [14–16]. In addition, cognitive deficits appear to be independent of the severity of positive symptoms and are only mildly correlated with the severity of negative symptoms [17]. All of which suggests that cognitive deficits in schizophrenia have a different underlying pathological process than those underlying the clinical symptoms of the disorder [18].

Bearing in mind that schizophrenia involves, at least in part, prefrontal cortex dysfunction [19,20], and that this cognitive deficit has been strongly related to real-world functioning in the disorder [21,22], it is not surprising that research on the study of CFT in schizophrenia has increased significantly in recent years. Accordingly, in keeping with the content-specific pathway of CFT, research to date has found global disruption in counterfactual reasoning in Step 1 and Step 2 of the regulatory sequence. Interestingly, the implementation of corrective behaviours (Step 3) appears to be intact in these patients [23]. With regard to these findings, however, it should be noted that although deficits in CFT activation (Step 1) have been widely reported not only in patients with schizophrenia [24–26] but in their unaffected first-degree relatives [27], research into the activation of behavioural intentions (Step 2) is still scarce [28].

Specifically, findings concerning the activation of behavioural intentions (Step 2) in schizophrenia are currently based on a single study carried out by Roese et al. in 2008, in which the facilitator effect of CFT on the activation of behavioural intentions was tested with a semantic priming task developed by these authors using reaction time (RT) as the dependent variable [28]. Fifteen patients with schizophrenia and 13 healthy subjects performed 45 trials where they had to give a yes/no answer to a declaration of intention (i.e., the intention to carry out a specific action in the future). Each trial presented a negative event that was judged using a within-subject sequential priming paradigm in one of three ways: a counterfactual (“should have”), a neutral control (a word-counting judgement) or a no-judgement baseline. Results showed that whereas the healthy controls responded faster to counterfactual judgements relative to control judgements, the patients with schizophrenia’s RT did not vary across the different primes—i.e., the CFT trial did not facilitate the activation of behavioural intentions compared with the neutral control trial. The authors concluded that the link between CFT and the generation of behavioural intentions was broken in schizophrenia, stating that “counterfactuals did not activate intentions in patients with schizophrenia (p. 2)” and suggesting that rehabilitation strategies designed to normalize CFT could not have any benefit for these patients.

Roese et al’s study [28] would benefit from replication and extension. With this objective in mind, the present study modified the semantic priming task and evaluating the largest sample of patients with schizophrenia and healthy control subjects to date. We hypothesized that both study groups would commit fewer errors and would response faster when confronted with a counterfactual prime than when confronted with a neutral-control prime. It was hypothesized that schizophrenia patients would perform more poorly than the healthy control subjects. If demonstrated, the latter finding suggest the possibility of targeting CFT in future treatment approaches. In addition, potential associations with variables of neurocognition, clinical status and socio-demographic characteristics were explored in the study.

## Method

### Participants

Seventy-four participants (37 patients with schizophrenia and 37 healthy control subjects) all fluent in Spanish and aged between 19 and 68 were included in the study after an initial inclusion interview in which mental and personality disorders were assessed using the Structured Clinical Interview for DSM-IV Axis I Disorders (SCID-I) [29] and Axis II Personality Disorders (SCID-II) [30]. The Clinical Research Ethics Committee of Bellvitge University Hospital (CEIC) approved all study procedures, and all subjects gave written informed consent before inclusion.

All patients with schizophrenia were recruited from the outpatient service of the Psychiatry Department of Bellvitge University Hospital, met DSM-IV-TR criteria [31] and had not undergone electroconvulsive therapy in the last six months. Patients with a diagnosis of bipolar, schizoaffective, delusional or other Axis I disorders were excluded. Healthy control participants were recruited from hospital employees; exclusion criteria were a previous history of personal or family psychiatric illness (Axis I and Axis II).

Participants were excluded if they had a history of substance use disorder as defined according to DSM-IV-TR [31] (with the only exception being nicotine dependence), head trauma involving loss of consciousness, neurological disease or medical illness that could affect brain function, or an estimated Intelligence Quotient (IQ) below 70. A one-to-one matching procedure was employed to match the control group with the schizophrenia patients by sex, age and educational level.

### Measures and procedure

Participants attended individual testing sessions lasting an average of four hours. Clinical rating scales, socio-demographic characteristics and cognitive tests were administered by experienced psychiatrists and neuropsychologists.

Socio-demographic and clinical variables were recorded using an in-house standardized medical history. Symptoms and severity of illness were assessed with the Spanish version of the Positive and Negative Syndrome Scale (PANSS) [32,33] and the Clinical Global Impression-Schizophrenia Scale (CGI-SCH) [34]. Level of functioning was assessed using the Global Assessment of Functioning scale (GAF) [35]. Pharmacological treatment was recorded, and antipsychotic (AP) daily dose equivalents of chlorpromazine were calculated [36].

The Spanish version of the vocabulary subtest of the Wechsler Adult Intelligence Scale III battery (WAIS-III) was used to calculate an estimated IQ [37]. Cognitive function was evaluated with the Spanish version of the Brief Assessment of Cognition in Schizophrenia (BACS) [38,39].

### Experiment: Activation of behavioural intentions

**Fig 1:**
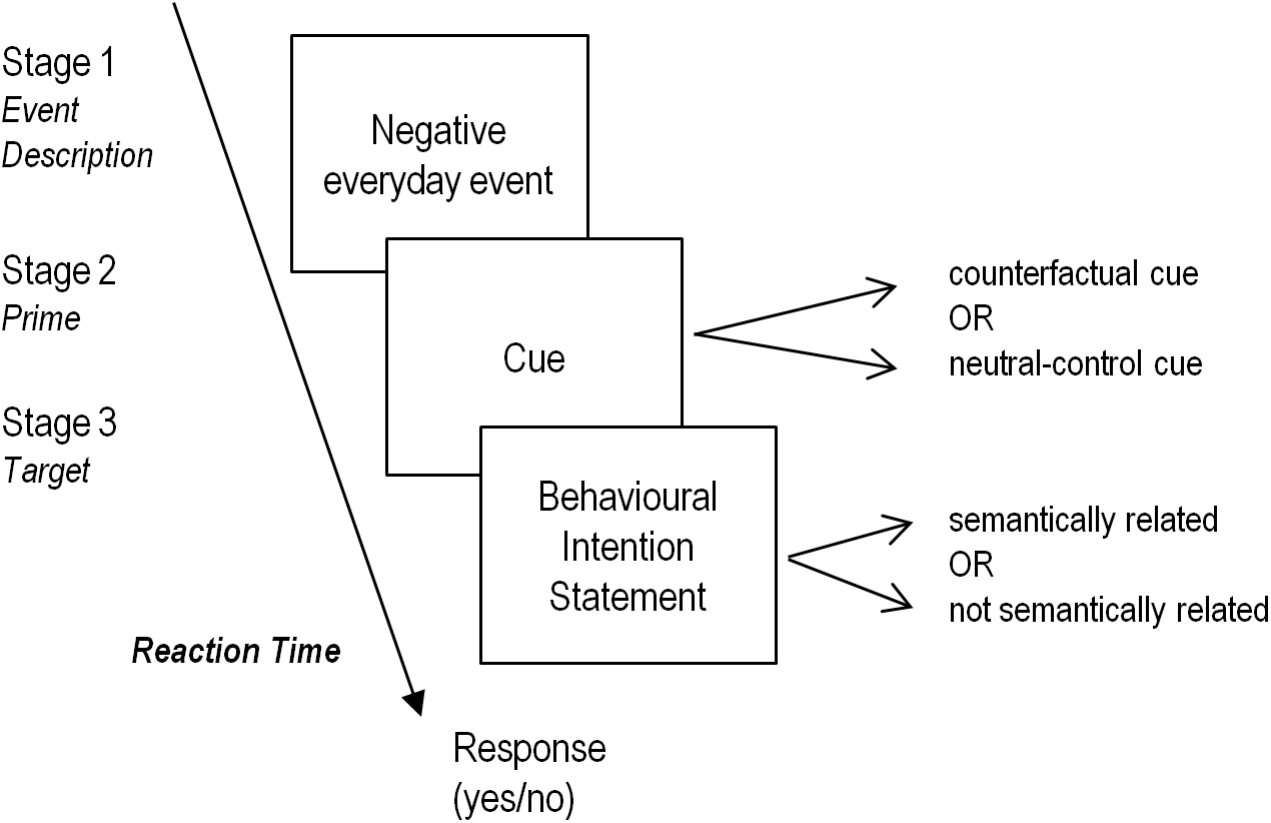
Overview of the sequential priming paradigm adapted from the original sequential priming paradigm designed by Roese et al. (2008) [28].

To assess whether a preceding counterfactual judgement facilitated the activation of a relevant behavioural intention judgement, an experiment was developed using an adaptation of the original sequential priming paradigm designed by Roese et al. [28] (see **Fig 1** for an overview of the procedure). Testing was implemented using desktop computers running DMDX software [40] and consisted of two blocks of 16 trials: first a 2-minute training block, followed by the 10-minute experiment block. The order of presentation of trials was randomized within each block across participants. In this way, all subjects completed 32 judgement trials that were structured in three stages around a hypothetical negative event: first, a description of a negative everyday event appeared on the screen (stage 1) (e.g., “I have missed the train”); two seconds later, a prime cue (stage 2) that could be a counterfactual statement (e.g., “I should have”) or a neutral-control statement (a factual-neutral cue such as “It has five words”) appeared. The neutral-control prime task was modified from the task used by Roese et al. (2008): while in the original experiment participants executed a word-counting judgement [28], in the current study the neutral-control task consisted of reading a statement, as in the counterfactual condition. This ensured that the cognitive load of the procedural work was similar in the two types of task. This stage was followed by a subsequent behavioural intention statement that was either semantically related to the action described previously or not (stage 3) (e.g., “got out of bed sooner” or “washed the car before”). Finally, participants had to press a key on the computer labelled “yes” or “no” to indicate whether the behavioural intention was related to the negative everyday event that preceded it.

Once the experiment was finished, two outcomes were recorded: (1) response to complete the target task (wrong or correct) understood as the associations made by the participants between the first event and the final outcome–e.g., answering “no” when the event and the intention judgement were actually related, or vice versa, and (2) percentage gain in the reaction time (RT) to achieve a correct association or answer whether the prime was a counterfactual or a neutral-control cue. Note that RT to achieve a correct association was defined as the time gap measured in milliseconds from stage 3 and participants’ response, and that percentage gain was defined as the difference between RT in the neutral-control prime and RT in the counterfactual prime divided by RT in the neutral-control cue. The decision to use percentage gain rather than raw RT scores was based on previous research findings highlighting a potential arithmetical artefact due to the effect of a general RT slowing in schizophrenia [41]. Specifically, the value for priming will be spuriously inflated in patients with the disorder, if they are slower to respond on both the unprimed and primed versions of the task [42].

### Statistical analysis

For descriptive analyses, absolute and relative frequencies were calculated for categorical variables. Continuous variables were assessed using the mean and standard deviation (SD) for normally distributed variables, and the median and interquartile range (IQR) for non-normally distributed variables. To detect differences between groups, Fisher’s exact test and χ^2^ were used for categorical data, whereas group means were compared using two-tailed Student’s T test and Wilcoxon rank sum test.

To evaluate the effect of the prime presented (counterfactual vs. neutral-control), two outcomes were examined. First, the adjusted odds ratio of a wrong association on schizophrenia patients versus healthy controls was estimated using a mixed logistic regression model. Socio-demographic and clinical variables were the potential adjusting variables tested. Secondly, the adjusted effect of being a schizophrenia patient versus a healthy control when assessing the percentage gain in the RT difference was estimated using a mixed regression model, with the same potential adjusting variables being tested. As percentage gain did not follow a normal distribution, a log transformation was performed. Normality was tested graphically by quantile plot and analytically by the Shapiro-Wilks test. For both models, a random subject effect was included to account intra-individual variability on both outcomes among all participants.

Furthermore, as the experiment included 16 repeated trials and a possible learning effect had to be considered, number of trial variable was included in the estimated models with no effect expected. In addition, as the learning effect could differ between patients and controls, the first interaction term was also evaluated. The selection of the model’s variables was based on the Akaike Information Criterion (AIC). Fixed effects were tested for statistical significance using the Wald test. Coefficients in the mixed regression model are presented in log scale and odds ratios in the mixed logistic model together with 95% confidence interval and p-values.

Finally, for both outcomes, a second model was estimated for the schizophrenia patients only; this was in order to explore the clinical measures included in the study. Thus, along with the aforementioned potential confounder variables, other clinical variables were included such as daily dose of AP taken (chlorpromazine equivalents) in mg/day, duration of the illness in years, and scores on the PANSS and GAF scales were tested to adjust the significance of the prime task effect. The analysis of residual and influential values did not identify any covariate pattern with a relevant impact on the goodness of fit statistics, or deviance residual, or in estimating the coefficients model. Data were managed and analyzed using R 3.2.5.

## Results

Socio-demographic characteristics are shown in **Table 1**. There were no differences regarding sex, age, educational level or hand dominance between patients with schizophrenia and healthy controls, but a larger proportion of patients were unemployed/retired and single at enrolment. With regards to neurocognitive performance, the patients had significantly lower scores in all cognitive domains in comparison with the healthy control subjects. No statistically significantly associations were found between any of the experimental measures and neurocognitive performance in either the healthy controls or the schizophrenia patients (these analyses are presented in more detail in **S1 Appendix**).

**Table 1:** Socio-demographic characteristics of the sample.

	Schizophrenia Patients (n = 37)	Healthy Controls (n = 37)	p-value
Male sex, *n (%)*	23 (62.16)	21 (56.76)	0.813^a^
Age (years)	38.49 (10.20)	40.12 (12.52)	0.542^b^
Educational level (years)	10.62 (3.46)	11.57 (3.11)	0.073^c^
Employment status, *n (%)*			0.000^d^
Employed	13 (35.14)	32 (86.49)	
Unemployed/ Retired	23 (62.16)	5 (13.51)	
Student	1 (2.70)	0 (0.00)	
Marital status, *n (%)*			0.020^d^
Single	30 (81.08)	19 (51.35)	
Married	6 (16.22)	15 (40.54)	
Divorced	1 (2.70)	3 (8.11)	
Hand Dominance (right), *(%)*	94.59	97.30	1.000^d^
Estimated IQ	97.57 (11.34)	112.03 (8.93)	0.000^b^

*Note*. Values presented as means (standard deviation) unless specified otherwise.

^a^ χ^2^ test

^b^ T-test

^c^ Wilcoxon rank sum test

^d^ Fisher’s Exact Test.

In terms of clinical characteristics, the patients exhibited mild levels of symptoms on the PANSS: total score = 74.24 (SD = 16.08), positive dimension = 13.59 (SD = 3.42), negative dimension = 22.35 (SD = 5.94) and general dimension = 38.30 (SD = 8.65).The median GAF score was 60 (IQR = 60–70), mean length of illness was 16.03 years (SD = 9.79) and the mean daily dose of AP treatment taken was 657.13 mg/day (SD = 470.17) (chlorpromazine equivalents).

### Experiment: Activation of behavioural intentions

Descriptive data on each prime task condition according to study group is presented in **Table 2**.

**Table 2:** Activation of behavioural intentions experiment.

	Counterfactual prime condition	Neutral-control prime condition
Incorrect associations made, *n (%)*		
Healthy controls	26 (4.4)	19 (3.2)
Schizophrenia patients	56 (9.5)	106 (17.9)
RT to correct association (ms), *median (IQR)*		
Healthy controls	1136 (855–1442)	1235 (941–1538)
Schizophrenia patients	1614 (1271–2059)	1808 (1404–2371)

*Note*. In both prime conditions, the number of observations was 592. ms: milliseconds.

#### Response to complete the target task (wrong or correctly): Mixed logistic regression model results

Results from the mixed logistic regression model are summarized in **Table 3**.

**Table 3:** Mixed effects logistic regression using right/wrong answer as the dependent variable.

	Entire sample model^a^ (n = 74)			Schizophrenia patients model^b^ (n = 37)		
	OR	CI 95%	p-value	OR	CI 95%	p-value
Constant	0.03	0.02 to 0.07	<0.0001	0.17	0.08 to 0.36	<0.0001
HC vs. SCZ	3.89	1.99 to 7.58	<0.0001	—		
NC vs. CFT	0.58	0.42 to 0.79	0.001	0.41	0.40 to 0.43	<0.0001
Number of trial	0.96	0.93 to 0.99	0.027	0.95	0.49 to 1.84	0.008
Age (centered)	1.01	0.98 to 1.04	0.468	1.02	0.52 to 2.02	0.654
Sex, male	1.49	0.75 to 2.95	0.250	1.29	0.94 to 1.78	0.615
Educational level (centered)	0.93	0.84 to 1.03	0.183	0.92	0.89 to 0.95	0.273

OR: odds ratio; SCZ: schizophrenia patients; HC: healthy controls; NC: neutral-control prime condition; CFT: counterfactual prime condition.

^a^ 2368 observations in 74 clusters.

^b^ 1188 observations in 37 clusters.

For the *entire sample model*, the odds of responding incorrectly were significantly higher (1.7 times) when a neutral-control prime was presented. Among patients, the odds were 3.9 times higher than among controls after controlling for age, sex and education level. Moreover, a learning effect was found, i.e., for each new trial presented, the odds of making a wrong association were 4% lower. No significant interaction effect was found between type of prime task, study group or number of trials presented.

For the *schizophrenia patients model*, the odds of responding incorrectly were significantly higher (2.4 times) when a neutral-control prime was presented after controlling for age, sex and educational level. Additionally, a learning effect was observed, i.e., for each new trial presented, the odds of making a wrong association were 5% lower. No significant interaction effect was found between type of prime task, study group or number of trials presented. No clinical potential confounder variables presented a significant association, nor improved the model performance.

#### Percentage gain in the reaction time difference: General linear mixed model results

Results from the general linear mixed model regarding percentage gain in the RT difference are presented in **Table 4**.

**Table 4:** General linear mixed regression model using percentage gain difference to a right answer as the dependent variable.

	Entire sample model^a^ (n = 74)			Schizophrenia patients model^b^ (n = 37)		
	beta	CI 95%	p-value	beta	CI 95%	p-value
Constant	4.50	4.48 to 4.60	<0.0001	4.50	4.41 to 4.60	0.000
HC vs. SCZ	-0.04	-0.09 to 0.00	0.070	—		
Number of trial	-0.00	-0.01 to 0.00	0.354	-0.01	-0.01 to 0.00	0.170
Age (centered)	0.00	-0.00 to 0.004	0.082	0.01	-0.00 to 0.01	0.143
Sex, male	0.01	-0.04 to 0.06	0.758	0.03	-0.05 to 0.11	0.451
Educational level (centered)	0.00	-0.00 to 0.01	0.167	0.01	-0.01 to 0.01	0.761

*Note*. Family Gamma using log as link function. SCZ: schizophrenia patients; HC: healthy controls; NC: neutral-control prime task; CFT: counterfactual prime task.

^a^ 994 observations in 74 clusters.

^b^ 444 observations in 37 clusters.

As noted above, percentage gain was measured by dividing the raw reaction time difference by the reaction time to a correct answer under a neutral-control prime. However, to calculate this it was necessary that in each trial the subject answered correctly in both prime conditions. Hence, if a subject answered correctly under the counterfactual prime but not under the neutral-control prime, or vice versa, the percentage gain was considered as missing. Consequently, taking in account that the percentage of trials presenting a correct response in both prime conditions was 76% among schizophrenia patients and of 93% among controls, the final available sample for the percentage gain model consisted of 994 trials over 1184 potential trials (16 trials in each prime in each group of study).

After adjusting for age, sex and educational level no significant difference between patients and controls was in percentage gain. It should be noted that the expected percentage gain difference in an average participant corresponds to the log of the model's intercept. The percentage gain of a random participant, independently of the study group, was 8%–i.e., the RT when a counterfactual prime was presented was 8% lower in relation to the RT when a neutral-control prime was presented. Accordingly, percentage gain of a random patient was 9% after adjusting for age, sex and educational level in the *schizophrenia patients model*. None of the clinical potential confounder variables assessed presented a significant association.

Finally, for both models, no learning effect was found in this measure across trials in either group.

## Discussion

The present study focused on the activation of behavioural intentions facilitated by CFT–the capacity of inferring how an event might have unfolded differently in response to real-world experiences [43]–in the largest sample of patients with schizophrenia and healthy control subjects examined to date. In schizophrenia, it has been proposed that this specific counterfactual skill is disabled [28]. However, these results come from a single study with some methodological concerns; therefore, the present study was designed to re-evaluate these previous findings, using a larger sample and an improved priming paradigm. In addition, potential associations with variables of neurocognition, clinical status and socio-demographic characteristics were explored. Several results were found that merit discussion.

In the first place, it was found that both the patients and the controls made fewer errors and performed faster in the counterfactual semantic priming condition than in the neutral-control priming condition. Hence, the present results support our main hypothesis that patients with schizophrenia would show a counterfactual semantic facilitation, as healthy controls do. Accordingly, as well as confirming previous research in healthy subjects [6], the present study does not support the claim that activation of behavioural intentions facilitated by CFT is disabled in schizophrenia. In fact, as evidenced by a fall in the odds of answering incorrectly (i.e., response to complete the target task variable), the patients with schizophrenia in this study were capable of learning from experience as the experiment progressed.

One possible explanation for the difference between our findings and those of Roese et al. [28] might be that the sample used in our study (37 patients and 37 controls) was larger than the one used in their study (15 patients and 13 controls). Another explanation might be related to the adaptation made to the original experiment involving changes to the neutral-control prime condition. In Roese et al.’s [28] control condition the participants executed a word-counting judgement which represented a task with significant cognitive demands, and was not comparable to the counterfactual condition task which consisted only of reading a statement. To rectify this problem, in the present experiment the neutral-control condition was modified so that it consisted simply of reading a statement focused on a factual cue rather than executing a cognitive task. The level of difficulty was more similar to that of the counterfactual trial, and the design additionally ensured that participants did not know a priori whether the third message was related to the first event until it appeared on the computer screen.

Secondly, although to our knowledge this is the first time that the potential associations between CFT facilitation of behavioural intentions and clinical, socio-demographic or cognitive measures have been explored, these analyses did not reveal evidence of significant associations with any of these variables. Indeed, the relationship between low and high order cognitive processes is controversial. For instance, whether social cognition and basic cognitive processes are associated is a question not yet adequately answered [44–45]. Thus, similarly, the debate about the observed counterfactual disruption in schizophrenia is the result of a pervasive cognitive impairment or is dependent on a specific deficit in a certain cognitive domain can still be considered to be open. Further research using other neuropsychological measures, for instance, instruments assessing domains of social cognition, might be of interest.

The present study has some limitations that should be acknowledged. Although the sample used was larger than in the one previous study [28], the number of participants remained relatively small. This may have resulted in a lack of statistical power and a type II error, raising the possibility that the study was not able to detect actual differences between groups. Secondly, in order to avoid a potential effect of greater cognitive deterioration in clinical and neurocognitive measures among older schizophrenic patients, healthy subjects were matched by age, sex and educational level, and all analyses were adjusted for these same variables. Thirdly, the study sample did not meet the criteria for stability as defined by Andreasen et al. (2005) [46], although the mean total PANSS score was 74.24 (SD = 16.08) which indicates a relatively low level of current symptoms. Finally, two issues about the experimental design have to be considered: first, this experiment was designed without a baseline (no judgement) condition and second, although the instruction for the participants was to only read the statements, the neutral-control (factual) cues presented (for instance, “it has 5 words”) might be counterintuitive for the participants. Further research including these considerations could help to achieve a clearer assessment of the effect of CFT on behavioural intentions activation.

## Conclusions

In summary, the findings of this study suggest that patients with schizophrenia preserve their capacity to generate intentions as a precursor to behavioural implementation once CFT is activated. These findings indicate that, in spite of the conclusions of previous research, Step 2 of the CFT content-specific pathway is actually not broken in the disorder. Therefore, in the light of previous studies demonstrating the feasibility of cognitive bias modification programs in the psychotic population [47–49], the present findings may be of interest since from the perspective of targeting counterfactual reasoning deficits in future treatment approaches in order to improve these patients’ reasoning and functional outcome. If, patients with schizophrenia can produce behavioural intentions facilitated by counterfactual judgements, they may also benefit from a specifically cognitive rehabilitation treatment focused on the understanding of negative experiences and the activation of the corresponding corrective intentions. This might help them to regulate and improve their behaviours as well as their future functioning. Further confirmatory studies are needed in order to corroborate the present results.

## Supporting information

S1 Appendix Neurocognitive performance and its association with results from the experiment. (PDF)
