# Supplementary material for: Patients with schizophrenia activate behavioural intentions facilitated by counterfactual reasoning
Source: PLoS One. 2017 Jun 6;12(6):e0178860. doi: 10.1371/journal.pone.0178860 (PMC5460856; doi:10.1371/journal.pone.0178860)
Supplement: S1 Appendix — (PDF) [file pone.0178860.s001.pdf]

S1 Appendix. *Neurocognitive performance and its association with results from the experiment*

As presented in **Table S1**, schizophrenia patients obtained significantly lower scores in all cognitive domains than the healthy control subjects.

Table S1. Neurocognitive performance by groups of study.

	Schizophrenia Patients (n=37)	Healthy Controls (n=37)	p-value
**BACS, raw scores**			
Verbal memory	34.39 (12.27)	44.16 (6.07)	0.000[b]
Digit sequence	14.62 (4.18)	18.30 (3.38)	0.000[b]
Token motor task	69.08 (15.51)	83.65 (9.14)	0.000[a]
Verbal fluency	28.54 (10.29)	44.51 (8.56)	0.000[a]
Symbol coding	36.22 (14.04)	54.32 (8.39)	0.000[b]
Tower of London	17.81 (2.91)	19.08 (1.66)	0.033[b]

Note. Values presented as means (standard deviation) unless specified otherwise.
[a]T-test; [b]Wilcoxon rank sum test; BACS: The Brief Assessment of Cognition in Schizophrenia.

Furthermore, no significant associations were found between any of the cognitive variables assessed and none of the both outcomes measures in the experiment.

This total lack of association with cognitive functioning is not surprising since inferring from CFT, as a higher order process, involves more complex reasoning than other low order cognitive functions such as attention or working memory. Along with the fact that previous research has not found significant correlations between activation of CFT and any of the basic cognitive deficits characteristic of schizophrenia [1–3]. Present results are also supported by recent neuroimaging studies' findings proposing an integrative network of systems for affective processing, mental simulation and cognitive control in which CFT might be relying on. This proposal considers that

counterfactual reasoning might actually be supported by the coordination of multiple information processing systems that together enable adaptive behavior [4, 5, 6]. Thus, further research could benefit from also including neuroimaging measures other than neuropsychological.

References

1. Hooker C, Roese NJ, Park S (2000) Impoverished counterfactual thinking is associated with schizophrenia. Psychiatry 63: 326–335.

2. Contreras F, Albacete A, Castellví P, Caño A, Benejam B, et al. (2016) Counterfactual reasoning deficits in schizophrenia patients. PLoS One 11: e148440.

3. Albacete A, Contreras F, Bosque C, Gilabert E, Albiach Á, et al. (2016) Counterfactual reasoning in non-psychotic first-degree relatives of people with schizophrenia. Front Psychol 7:665.

4. Van Hoeck N, Watson PD, Barbey AK (2015) Cognitive neuroscience of human counterfactual reasoning. Front Hum Neurosci 9.

5. Van Hoeck N, Ma N, Ampe L, Baetens K, Vandekerckhove M, et al. (2012) Counterfactual thinking: an fMRI study on changing the past for a better future. Soc Cogn Affect Neurosci 8: 556–564.

6. Barbey AK, Krueger F, Grafman J (2009) Structured event complexes in the medial prefrontal cortex support counterfactual representations for future planning. Philos Trans R Soc Lond B Biol Sci 364: 1291–1300.
